# Crystal Structure and Noncovalent Interactions of Heterocyclic Energetic Molecules

**DOI:** 10.3390/molecules27154969

**Published:** 2022-08-04

**Authors:** Yan Liu, Jiake Fan, Zhongqing Xue, Yajing Lu, Jinan Zhao, Wenyan Hui

**Affiliations:** 1 Department of Environmental and Safety Engineering, Taiyuan Institute of Technology, Taiyuan 030008, China; 2 School of Environment and Safety Engineering, North University of China, Taiyuan 030051, China

**Keywords:** heterocyclic compounds, crystal structure, stacking mode, intermolecular interactions

## Abstract

Nitrogen-rich heterocyclic compounds are important heterocyclic substances with extensive future applications for energetic materials due to their outstanding density and excellent physicochemical properties. However, the weak intermolecular interactions of these compounds are not clear, which severely limits their widespread application. Three nitrogen-rich heterocyclic compounds were chosen to detect their molecular geometry, stacking mode and intermolecular interactions by crystal structure, Hirshfeld surface, RDG and ESP. The results show that all atoms in each molecule are coplanar and that the stacking mode of the three crystals is a planar layer style. A large amount of inter- and intramolecular interaction exists in the three crystals. All principal types of intermolecular contacts in the three crystals are N···H interactions and they account for 40.9%, 38.9% and 32.9%, respectively. Hydrogen bonding, vdW interactions and steric effects in Crystal **c** are stronger than in Crystals **a** and **b**. The negative ESPs all concentrate on the nitrogen atoms in the three molecules. This work is expected to benefit the crystal engineering of heterocyclic energetic materials.

## 1. Introduction

Energetic materials (EMs), mainly including explosives, propellants and pyrotechnics are a class of important metastable compounds that involve explosive groups, or oxidants and reducers, that can transiently release considerable energy through their self-redox reactions after sufficient stimulation and have occupied an important place in mining, military equipment, space exploration and fireworks [1,2,3,4,5,6,7,8]. Regarding an EM, energy and safety, which significantly depend on the crystal packing, are two of the most important concerns and attract the most attention, as the energy represents their efficiency and the safety guarantees their applicability. Therefore, the properties and performances, such as density, energy, safety, mechanical properties, environmental adaptability, aging performance and so on, should be strictly and comprehensively evaluated for the design and development of EMs.

Heterocyclic energetic compounds have been identified as promising contenders to traditional EMs. They possess outstanding density, higher formation enthalpy, excellent stability and attractive performance and have extensive future applications in the field of energetic materials [9,10,11,12,13]. Nevertheless, the weak intermolecular interactions of heterocyclic energetic materials remain unclear, which limits their preparation and hinders their expansion in the field of energetic materials [14]. The relationship among component, molecular structure and chemical property is the core issue of materials science. That is, molecules, which are composed of a diversity of atoms, possess their inherent characteristics and not only depend on the type of atoms but also on the way in which they are connected [15]. The characteristic properties of molecules are influenced to a great extent by their organizational form when the atoms are selected.

It is well known that the coincidence of many chemical processes often occurs through a combination of various noncovalent interactions [16,17,18]. Molecules interacting with other molecules can occur through weak interactions (0.1–5 kcalmol^−1^), such as hydrogen bonds, van der Waals forces, halogen bonds, lithium bonds, π-π stacking, anions-π, cations-π, σ-σ, and lone pairs-π, which are collectively called noncovalent interactions and usually hidden within voids in the bonding network [15]. The dominance of noncovalent interactions is so strong that they resemble covalent bonds in some moments during chemical processes [19,20]. Intermolecular interaction is a feasible means to tune the properties of molecular structure [21,22,23]. Hence, a major challenge for designing excellent heterocyclic energetic compounds is to clarify the crystal structure and the intermolecular interactions.

With respect to some special properties and performances, heterocyclic energetic compounds are exciting and desired. Obviously, clarifying the relationships in heterocyclic energetic compounds will facilitate the application of heterocyclic energetic materials. In the present work, we focus on the molecular structure, stacking mode and intermolecular interactions by employing three nitrogen-rich heterocyclic energetic molecules, 5,6-dihydrotetrazolo [1,5-*c*]-quinazoline (C_8_H_7_N_5_, Molecule **a**), 4-azidopyrido [2,3-*d*] [1,2,3]triazine 2-oxide (C_6_H_3_N_7_O, Molecule **b**) and 3-tetrazolylpyrazin-2-nitramide hydrate (C_5_H_6_N_8_O_3_, Molecule **c**), extracted from the Cambridge Crystallographic Data Centre (CCDC) for analysis. In this highlight, we study the molecule geometry structure, two-dimensional (2D) fingerprint plots, Hirshfeld surface, reduced density gradient (RDG) isosurface, non-covalent interactions and electrostatic potential (ESP) surface to detect the intermolecular interactions. Hopefully, this work will deepen the understanding of heterocyclic energetic materials and provide a better insight for designing new EMs along the concept of crystal engineering.

**Figure 1:**
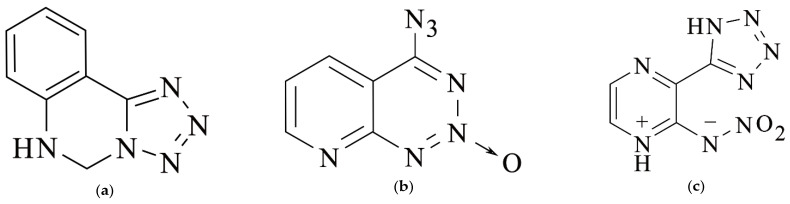
The chemical diagrams of three heterocyclic energetic molecules: (**a**) molecule **a**; (**b**) molecule **b**; (**c**) molecule **c**.

The chemical diagrams of the three heterocyclic energetic molecules are shown in Figure 1.

## 2. Materials and Methods

### 2.1. Materials

First, the data of molecular structure should be taken as a base to for the analyses and calculations to deepen the insight into the heterocyclic energetic molecules. As mentioned above, this work focuses on three existing heterocyclic energetic molecules. So, the objects, including 5,6-dihydrotetrazolo [1,5-*c*]-quinazoline [Molecule **a**, CCDC 2035798, Cambridge Structural Database (CSD) refcode EHAYUK], 4-azidopyrido [2,3-*d*] [1,2,3]triazine 2-oxide (Molecule **b**, CCDC 2035802, CSD refcode EHAZIZ) and 3-tetrazolylpyrazin-2-nitramide hydrate (Molecule **c**, CCDC 2035804, CSD refcode EHAZUL), were searched in the CCDC and saved as CIF files. The crystallographic parameters of three energetic crystals are shown in Supplementary Materials, Table S1 [13].

### 2.2. Methods

The intermolecular interactions of Crystals **a**, **b** and **c** were studied by Hirshfeld surface analysis, reduced density gradient (RDG) analysis and electrostatic potential (ESP) analysis, which provide an elegant way to detect the intermolecular interactions.

In a crystal, Hirshfeld surface analysis is a straightforward tool to distinguish and visualize the frequency, types and regions of intermolecular interactions [24]. Hirshfeld surfaces with their 2D fingerprints were created based on electron distributions and calculated as the sum of spherical atom electron densities using CrystalExplorer 21.5 [25]. The normalized contact distance (*d*_norm_) mapped on the Hirshfeld surfaces can be determined by *d*_i,_ *d*_e_, *r*_i_^vdw^ and *r*_e_^vdw^. The factors *d*_i,_ and *d*_e_ are the distances from the Hirshfeld surface to the nearest nucleus interior and exterior to the surface, respectively and *r*_i_^vdw^ and *r*_e_^vdw^ are the van der Waals (vdW) radius of the atom inside and outside the surface, respectively. Here, *d*_norm_ can identify the important information of intermolecular interactions. Projecting the point of (*d*_i_, *d*_e_) to the map obtains a 2D fingerprint plot that provides a visual summary of the relative frequencies of different intermolecular interactions. Both molecule geometry and intermolecular interaction are received from the Hirshfeld surface. In addition, the red dots on the visualized surface denote *d*_norm_ is negative and the intermolecular contacts are shorter than the van der Waals (vdW) radius. On the contrary, positive *d*_norm_ and longer intermolecular contacts are blue dots. When the value of *d*_norm_ is zero, the colour is white on the Hirshfeld surface.

RDG analysis is a powerful tool for molecular transformation and crystal stability, and it can also comprehensively study the influence of inter- and intramolecular interactions on crystal packing [26,27]. Based on electron density, the corresponding noncovalent interactions (NCI) plot was calculated in real space. In this method, the multiplication of $\mathrm{sign}\left(\lambda_{2}\left(r\right)\right)$ and ρ(r) gets the $\mathrm{sign}\left(\lambda_{2}\right)\;\rho$ function, which is projected onto the isosurface to visualize the location, strength and type of intermolecular interactions. The RDG will obviously change after a weak inter- or intramolecular interaction appears. So, hydrogen bonds, van der Waals interactions and repulsive steric clashes can be obtained from the colour filled RDG isosurface through probing the relationship between the quantum mechanical electron density (ρ) and RDG. The blue colour represents hydrogen bond regions, green represents vdW interaction regions and red represents steric regions. All RDG isosurfaces and NCI plots were calculated using Multiwfn and visualized using VMD [28,29].

ESP is an integral physical property of compounds generally. It can depict the dimensions and overall charge distribution and plays a key role in intermolecular interactions [30]. The ESP surface is a significant tool to study the information about charge density distribution and chemical reactivity sites on molecular surfaces, in which pivotal surface local minima and maxima of ESP are indicated as cyan and orange spheres, respectively, and the significant positive and negative of ESPs are labelled with the unit of kcal/mol. Herein, ESP analysis was used to study the electronic properties. The ESP surface of Crystals **a**, **b** and **c** was calculated by Multiwfn (Beijing Kein Research Center for Natural Sciences, Beijing, China) and visualized using VMD (University of Illinois, Champaign, IL, USA) [28,29].

## 3. Results

### 3.1. Crystal and Molecular Structure

**Figure 2:**
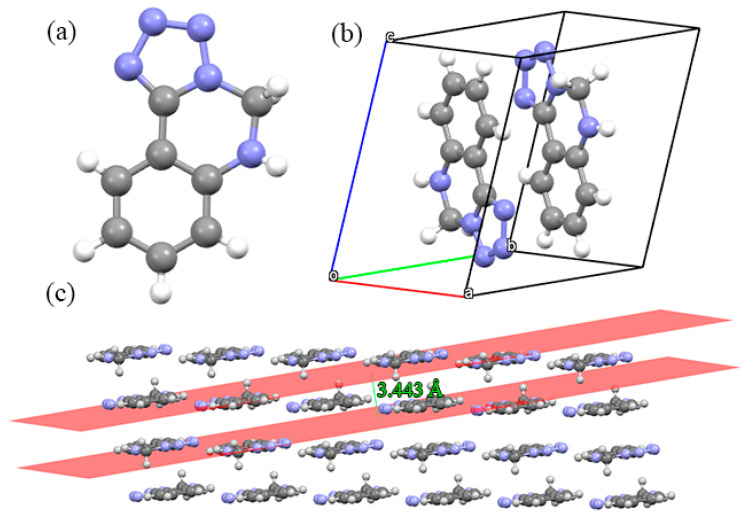
Crystal structures and geometrical configuration of Crystal **a**: (**a**) crystal structure; (**b**) single unit cell; (**c**) the 3D extended structure.

**Figure 3:**
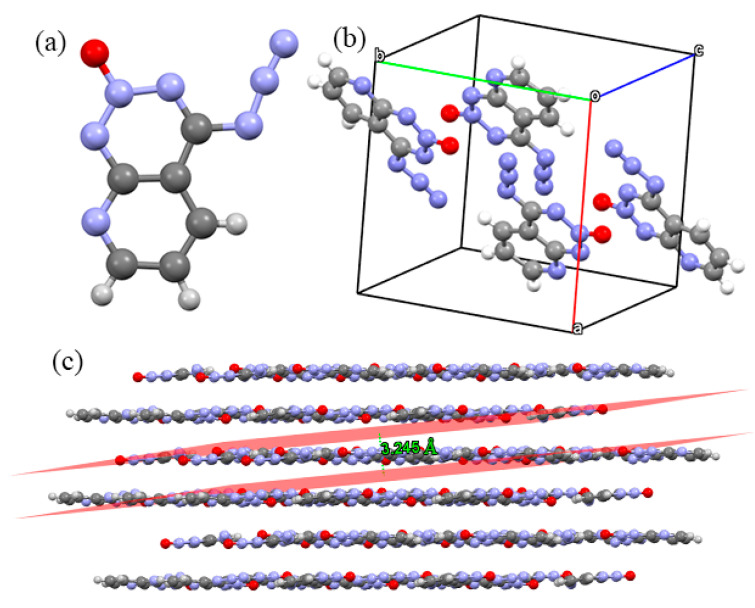
Crystal structures and geometrical configuration of Crystal **b**: (**a**) crystal structure; (**b**) single unit cell; (**c**) the 3D extended structure.

**Figure 4:**
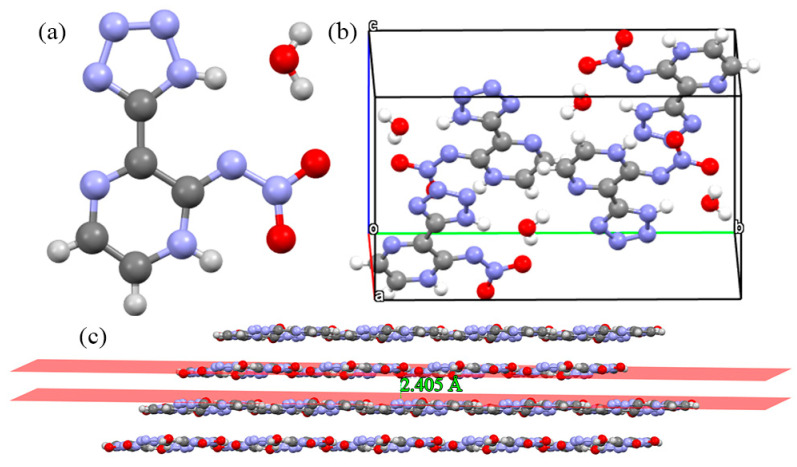
Crystal structures and geometrical configuration of Crystal **c**: (**a**) crystal structure; (**b**) single unit cell; (**c**) the 3D extended structure.

The morphology of a single crystal is a function of its lattice symmetry and the relative strength of the intermolecular interactions between molecules along different crystallographic directions [31]. The crystal structure and the geometrical configuration of Crystals **a**, **b** and **c** are shown in Figure 2, Figure 3 and Figure 4, respectively. In Figure 2a, the crystal structure of Crystal **a** is composed of two six-membered rings (a benzene aromatic ring and a quinazoline ring) and a five-membered ring (a tetrazole ring), where the rings are the skeleton to maintain the stability of the molecule. In Figure 2b, it is noticed that in Crystal **a** there are two molecules and that they are inverted between the head and tail in a unit cell. In addition, three rings of Crystal **a** in the skeleton are coplanar, which can be seen from their packing structure in Figure 2c. Crystal **a** contains planar ring structures and involves planar conjugated molecular structures. The planar conjugated molecular structures give π···π stacking interactions a sound basis and are stable bricks for building insensitive energetic materials. The π···π stacking interactions are stable to mechanical stimuli, due to free interlayer sliding [32,33,34,35,36,37,38]. As a consequence, π···π stacking interactions are usually necessary to strengthen molecular stability. This stacking mode is similar to TATB (an attractive insensitive explosive), which also indicates Crystal **a** possesses a good safety performance [39]. In Figure 2c, three rings ultimately repeat in the crystal structure forming a unique architectural platform and the interlayer distance is 3.443 Å.

In Figure 3a, the crystal structure of Crystal **b** is composed of two six-membered rings. One only possesses a N atom and is pyridine ring. The other possesses a -CN_3_ group and is a triazine ring. The two rings are the skeleton to maintain the stability of the molecule, and both rings belong to an *N*-heteroaromatic ring. As can be seen in Figure 3b, there are four molecules per unit cell in Crystal **b**. Apart from two rings in the skeleton that are coplanar, which can be seen from their packing structure in Figure 3c, Crystal **b** contains planar ring structures and involves planar conjugated molecular structures. The planar conjugated molecular structures give π···π stacking interactions a sound basis and are stable bricks for building insensitive energetic materials. The π···π stacking interactions are stable to mechanical stimuli, due to free interlayer sliding [32,33,34,35,36,37,38]. As a consequence, π···π stacking interactions are usually necessary to strengthen molecular stability. This layer-stacked crystal structure is similar to Crystal **a** and TATB, and also indicates a good safety performance. In Figure 3c, two rings ultimately repeat in the crystal structure creating a planar layer-stacked compound and the interlayer distance is 3.245 Å. 

In Figure 4a, the crystal structure of Crystal **c** involves two moieties (isomer3-tetrazolylpyrazin-2-nitramide and an H_2_O molecule). Isomer3-tetrazolylpyrazin-2-nitramide contains a pyrazine ring and a tetrazole ring as the skeleton, these rings maintain the stability of the molecule. In Figure 4b, we can see that in Crystal **c** there are four isomer3-tetrazolylpyrazin-2-nitramide molecules and four H_2_O molecules per unit cell. Apart from the isomer3-tetrazolylpyrazin-2-nitramide and H_2_O molecule that are coplanar, which can be seen from their packing structure in Figure 4c, Crystal **c** contains planar ring structures and involves planar conjugated molecular structures. The planar conjugated molecular structures give π···π stacking interactions a sound basis and are stable bricks for building insensitive energetic materials. The π···π stacking interactions are stable to mechanical stimuli, due to free interlayer sliding [32,33,34,35,36,37,38]. As a consequence, π···π stacking interactions are usually necessary to strengthen molecular stability. This layer-stacked crystal structure is similar to Crystals **a** and **b**, and TATB, and also indicates a good safety performance. In Figure 4c, two moieties (isomer3-tetrazolylpyrazin-2-nitramide and an H_2_O molecule) ultimately assemble in the crystal structure creating a planar layer-stacked compound and the interlayer distance is 2.405 Å. The above results show that the three crystals are planar layer-stacked compounds, and Crystal **c** possesses more compact crystal packing, due to a lower interlayer distance (2.405 Å).

### 3.2. Hirshfeld Surface 

**Figure 5:**
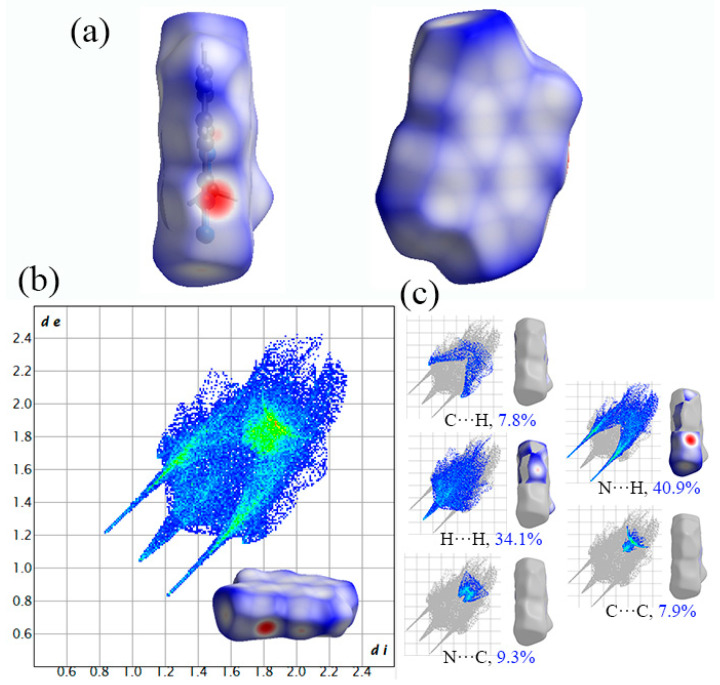
Hirshfeld surfaces and the corresponding 2D fingerprint plot of Crystal **a**: (**a**) Hirshfeld surfaces; (**b**) 2D fingerprint plots; (**c**) populations of close interatomic contacts.

To obtain a better understanding of how intermolecular interactions affect the crystal packing of Crystals **a**, **b** and **c** at the molecular level, the nature of the types, regions and percentage of intermolecular contacts was probed to quantify their contributions by Hirshfeld surfaces and corresponding 2D fingerprint plots. The Hirshfeld surface mapped with *d*_norm_ for Crystal **a** is depicted in Figure 5a, in which the geometry shape of the surface and the red dots thereon straightforwardly reflect the related stacking pattern and intermolecular interactions. It is noticed that the Hirshfeld surface is similar to a plate and red dots are distributed surrounding the edge of the plate, which suggests a planar layer stacking in Crystal **a**. Meanwhile, the red dots mean stronger and closer intermolecular contacts at the location of N, which indicates that N plays an important role during the chemical reaction in Crystal **a**. The 2D fingerprint plot is drawn according to *d*_i_ and *d*_e_ as horizontal and vertical coordinate axes, respectively, to a description of the types for intermolecular interactions. The shorter *d*_i_ + *d*_e_ of the spikes suggests a stronger interaction. In Figure 5b, a pair of remarkable spikes on the bottom left in the 2D fingerprint plots of the crystals denotes slightly stronger interaction among neighboring intralayer molecules. We can know that it is an N···H interaction and is the principal type of intermolecular contact, making the greatest contribution and accounting for the largest coverage portion (40.9%) of the total Hirshfeld surface in Crystal **a** (Figure 5c). N···H contacts can be C-H···N contacts on the two sides of the cycle moieties and are weak hydrogen bonds. The enrichment proportions of N···H contacts highlight that they turn out to be favored in the crystal packing. This also indicates that N and H play an important role during the chemical reaction in Crystal **a**. On the 2D fingerprint plot, the brightest part represents C···C interactions that are due to the six-membered C-ring of Crystal **a**. They are clearly discernible and account for 7.9% of the contribution among all interactions. Moreover, there are three other types of intermolecular contacts in Crystal **a**, including C···H, N···C and H···H interactions, and the contributions of these interactions are 7.8%, 9.3% and 34.1%, respectively. A larger proportion (34.1%) of H···H interactions indicates again the important role of H during the chemical reaction in Crystal **a**. On the other hand, C···H contacts are slightly favored and represent C-H···π stacking on the centre of the heterocycles in Crystal **a**. The results show that N and H play a very important role in Crystal **a**.

**Figure 6:**
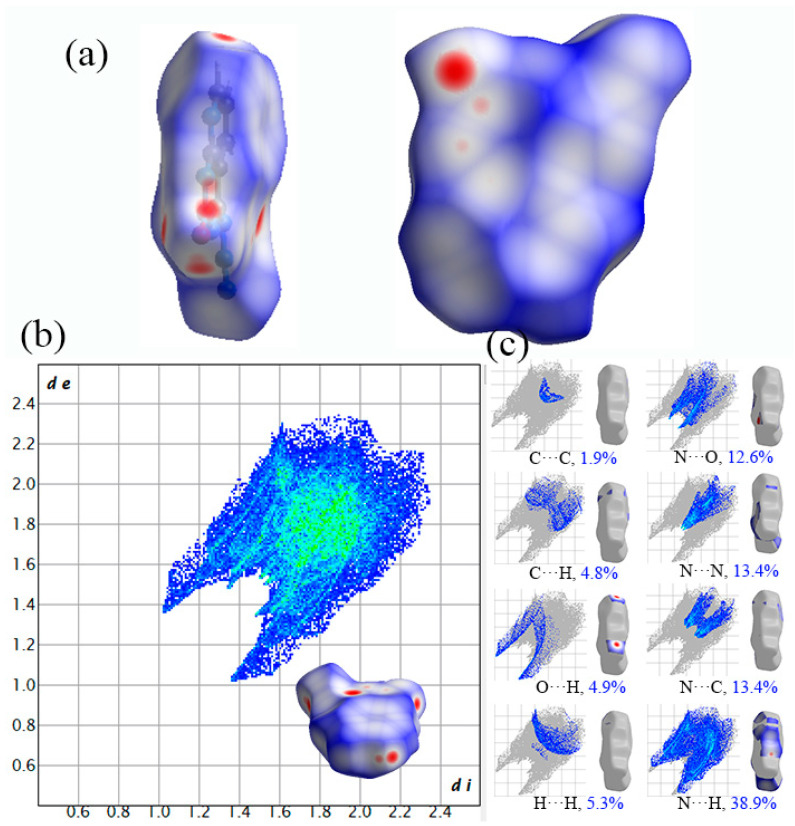
Hirshfeld surfaces and the corresponding 2D fingerprint plot of Crystal **b**: (**a**) Hirshfeld surfaces; (**b**) 2D fingerprint plots; (**c**) populations of close interatomic contacts.

The Hirshfeld surface mapped with *d*_norm_ for Crystal **b** is depicted in Figure 6a. As shown in Figure 6a, the Hirshfeld surface is also similar to a plate and red dots are distributed surrounding the edge of the plate, which suggests a planar layer stacking in Crystal **b**. Meanwhile, the red dots mean stronger and closer intermolecular contacts at the location of N and O, which indicates that N and O play an important role during the chemical reaction in Crystal **b**. The 2D fingerprint plot of Crystal **b** is shown in Figure 6b, in which a couple of distinctive spikes in the bottom left area of the wing are N···H interactions, and this is the principal type of intermolecular contact, making the greatest contribution and accounting for the largest coverage portion (38.9%) of the total Hirshfeld surface in Crystal **b** (Figure 6c). N···H contacts can be C-H···N contacts on the two sides of the cycle moieties and are weak hydrogen bonds. The enrichment proportions of N···H contacts highlight that they turn out to be favoured in the crystal packing. This also indicates that N and H play an important role during the chemical reaction in Crystal **b**. In addition, there are seven other types of intermolecular contacts in Crystal **b**, including C···C, C···H, O···H, H···H, N···O, N···N and N···C interactions, and the contributions of these interactions are 1.9%, 4.8%, 4.9%, 5.3%, 12.6%, 13.4% and 13.4%, respectively. In particular, C···H contacts are also slightly favoured and represent C-H···π stacking on the centre of the heterocycles in Crystal **b**. The results show that N, O and H play a very important role in Crystal **b**.

**Figure 7:**
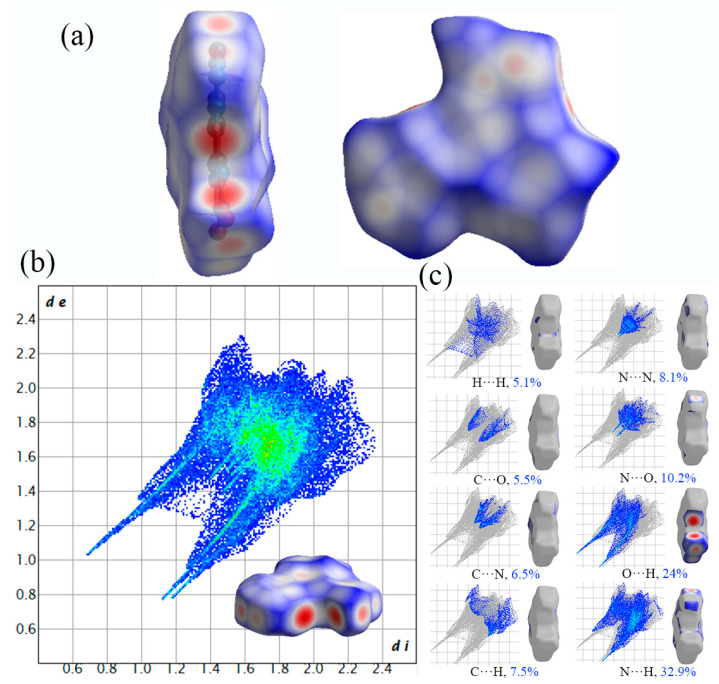
Hirshfeld surfaces and the corresponding 2D fingerprint plot of Crystal **c**: (**a**) Hirshfeld surfaces; (**b**) 2D fingerprint plots; (**c**) populations of close interatomic contacts.

The Hirshfeld surface mapped with *d*_norm_ for Crystal **c** is depicted in Figure 7a. As shown in Figure 7a, the Hirshfeld surface is also similar to a plate and red dots are distributed surrounding the edge of the plate, which suggests a planar layer stacking in Crystal **c**. Meanwhile, the red dots mean stronger and closer intermolecular contacts at the location of N and O, which indicates that N and O play an important role during the chemical reaction in Crystal **c**. The 2D fingerprint plot of Crystal **c** is shown in Figure 7b, in which a couple of distinctive spikes in the bottom left area of the wing are O···H and N···H interactions; these are the principal types of intermolecular contact, make an important contribution and account for coverage portions of 24% and 32.9%, respectively, of the total Hirshfeld surface in Crystal **c** (Figure 7c). N···H contacts can be C-H···N contacts on the two sides of the cycle moieties and are weak hydrogen bonds. The enrichment proportions of N···H contacts highlight that they turn out to be favoured in the crystal packing. This also indicates that N, O and H play an important role during the chemical reaction in Crystal **c**. In addition, there are six other types of intermolecular contacts in Crystal **c**, including H···H, C···O, C···N, C···H, N···N and N···O interactions, and the contributions of these interactions are 5.1%, 5.5%, 6.5%, 7.5%, 8.1% and 10.2%, respectively. Thereinto, C···H contacts are slightly favoured and represent C-H···π stacking on the centre of the heterocycles in Crystal **b**. The results show that N, O and H play a very important role in Crystal **c**.

### 3.3. Reduced Density Gradient Analysis

**Figure 8:**
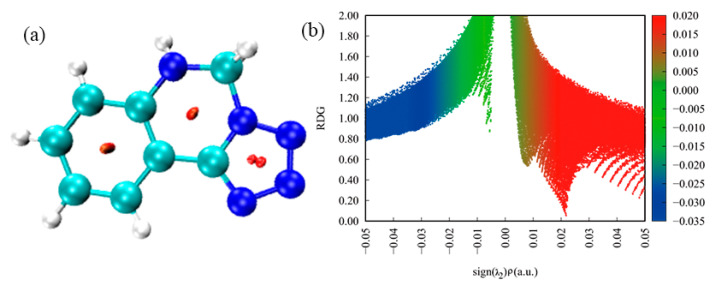
RDG map and NCI plot of gradient isosurface of Crystal **a**: (**a**) noncovalent interactions analyses; (**b**) scatter graph.

Apart from Hirshfeld surfaces, the colour filled (RDG) isosurface was performed in real-space based on the electron density to gain more information about the influence of the inter- and intramolecular interactions in the crystal structure by analysis of different colour regions. The surfaces are coloured on a blue–green–red scale based on values of $\mathrm{sign}\left(\lambda_{2}\right)\;\rho$ indicating strong attractive interactions, weak attractive and strong nonbonded overlap, respectively. As shown in Figure 8a, there is one dark red elliptical slab in the centre of each six-membered ring and there are two dark red elliptical slabs in the centre of the five-membered ring, indicating, in Crystal **a**, a stronger repulsion in the five-membered ring than in the six-membered ring. In Figure 8b, the blue region is a slender band at −0.05 < $\mathrm{sign}\left(\lambda_{2}\right)\;\rho$ < −0.03, which indicates that there are strong intermolecular interactions, such as hydrogen bonds in Crystal **a**. When −0.01 < $\mathrm{sign}\left(\lambda_{2}\right)\;\rho$ < −0.01, there is a green region that signifies weak intermolecular interactions and indicates the van der Waals (vdW) interactions with lower electron densities. It can be seen that within the red region there is an obvious spike that represents a strong steric effect and agrees well with the three dark red elliptical slabs of the molecular rings in Figure 8a.

**Figure 9:**
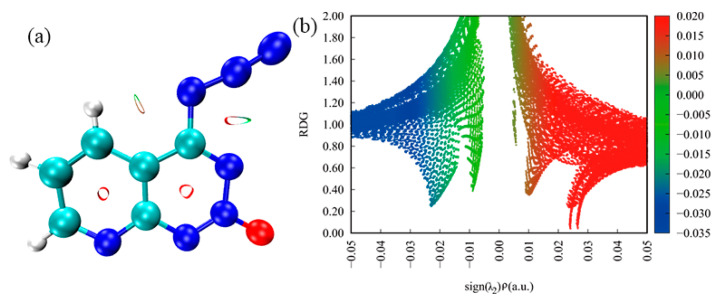
RDG map and NCI plot of gradient isosurface of Crystal **b**: (**a**) noncovalent interactions analyses; (**b**) scatter graph.

As shown in Figure 9a, four colourful circles are located in the NCI plot of Crystal **b**. It is of particular concern that a dark red circle is in the centre of each six-membered ring, indicating that a strong repulsion exists in the molecular structure of Crystal **b**. In Figure 9b, the blue region is a slender band at −0.05 < $\mathrm{sign}\left(\lambda_{2}\right)\;\rho$ < −0.03, which indicates that there are strong intermolecular interactions, such as hydrogen bonds, in Crystal **b**. When −0.03 < $\mathrm{sign}\left(\lambda_{2}\right)\;\rho$ < 0.01, there is a green region with two obvious spikes that signifies weak intermolecular interactions and belongs to the van der Waals (vdW) interactions with lower electron densities. It can be seen that during the red region, there are two obvious red spikes that represent a strong steric effect and agree well with the two dark red circles of molecular rings in Figure 9a. In addition, we can know that the vdW interactions in Crystal **b** are stronger than in Crystal **a** whereas the steric effects in Crystal **b** are weaker than in Crystal **a**.

**Figure 10:**
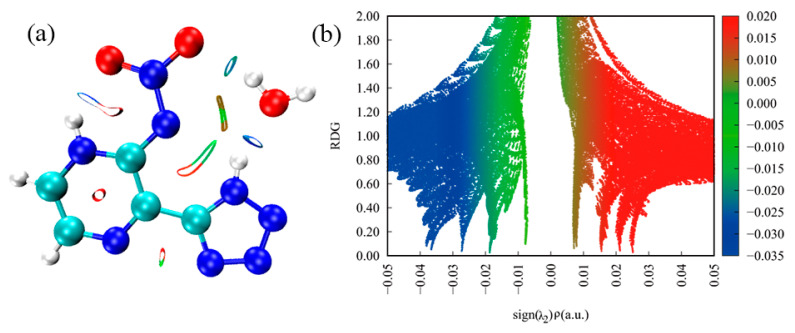
RDG map and NCI plot of gradient isosurface of Crystal **c**: (**a**) noncovalent interactions analyses; (**b**) scatter graph.

As shown in Figure 10a, seven colourful circles are located in NCI plot of Crystal **c**. It is of particular concern that a dark red circle is in the centre of the six-membered ring, indicating that a strong repulsion exists in the molecular structure of Crystal **c**. In Figure 10b, the blue region has two spikes at −0.05 < $\mathrm{sign}\left(\lambda_{2}\right)\;\rho$ < −0.02 that indicate that there is strong hydrogen bonding in Crystal **c**. When −0.02 < $\mathrm{sign}\left(\lambda_{2}\right)\;\rho$ < 0.01, there is a green region with three obvious spikes that signifies van der Waals (vdW) interactions. Within the red region, there are also three obvious red spikes that represent a strong steric effect and agree well with the dark red circles of the molecular rings in Figure 10a. Moreover, we can conclude that the intermolecular interactions involving hydrogen bonds, vdW interactions and steric effects, are stronger than in Crystals **a** and **b**.

### 3.4. Electrostatic Potential Analysis

**Figure 11:**
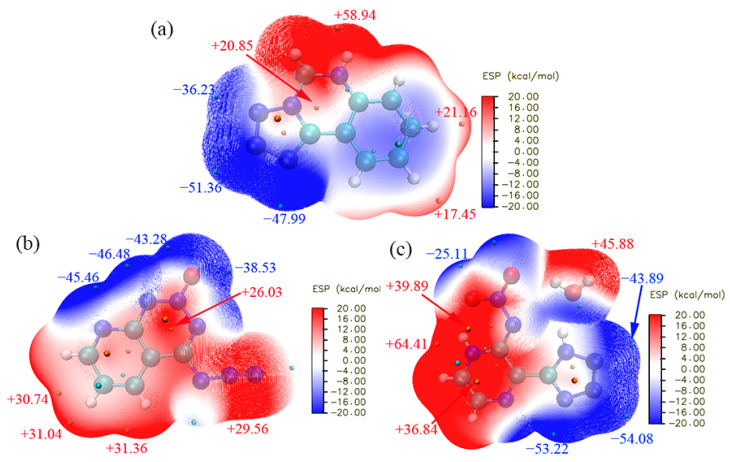
Electrostatic potential surfaces of Crystal **a** (**a**), Crystal **b** (**b**) and Crystal **c** (**c**).

To further understand the charge density distribution and molecular reactivity, the ESP of Crystals **a**, **b** and **c** was calculated based on the 0.001 electron/b^3^ isosurface of electron density. The result is shown in Figure 11. In Figure 11a, the stronger positive ESP of Crystal **a** is mainly located at one corner and the edge of the molecule, especially above the -NH bond, where the maximum of ESP is +58.94 kcal/mol. The negative ESP of Crystal **a** mainly concentrates on the nitrogen atoms of the five-membered ring and possesses a minimum of −51.36 kcal/mol. In Figure 11b, the stronger positive ESP of Crystal **b** is mainly located at the edge of the molecule, especially above the -CH bond, where the maximum ESP is +31.36 kcal/mol. The negative ESP of Crystal **b** mainly surrounds the nitrogen atoms of the six-membered ring and possesses a minimum of −46.48 kcal/mol. In Figure 11c, the stronger positive ESP of Crystal **c** is mainly located at the corner of the molecule, especially above the -OH bond of the H_2_O molecule and the −CH bond of the six-membered ring, where the maximum ESP is +64.41 kcal/mol. The negative ESP of Crystal **c** mainly surrounds the nitrogen atoms, especially around the five-membered ring and possesses a minimum of −54.08 kcal/mol. It can be seen from the above analysis that although the distribution area of positive ESPs is significantly different in the three crystals, the negative areas all concentrate at the nitrogen atoms of the three molecules. The transition area between positive ESP and negative ESP is a charge-neutral region, which is indicated by being coloured white. In Figure 11, there is a white transition zone in the three crystals signifying a large number of intermolecular interactions, which is well in accordance with Hirshfeld surface analyses. The electrostatic potential is a reliable criterion to evaluate the impact sensitivity and the impact sensitivity has a positive correlation with the surface potential maxima of the energetic materials^−^ [40,41,42,43]. The surface potential maxima of Crystals **a**, **b** and **c** are +58.94, +31.36 and +64.41 kcal/mol, respectively, indicating that the impact sensitivity of the three crystals is in the order Crystal **c** > Crystal **a** > Crystal **b**. Apart from impact sensitivity, the detonation velocity has a positive correlation with the sum of the surface potential maxima and minima of the energetic materials [44]. The sum of the surface potential maxima and minima of Crystals **a**, **b** and **c** are 7.58, −15.12 and 10.33 kcal/mol, respectively, indicating that the detonation velocity of the three crystals is in the order Crystal **c** > Crystal **a** > Crystal **b**.

## 4. Conclusions

In this paper, the characteristics of three nitrogen-rich heterocyclic compounds have been studied, including the molecular structure, stacking mode and intermolecular interactions. The results show that Crystal **c** possesses a more compact crystal packing than Crystals **a** and **b**, due to a lower interlayer distance (2.405 Å). All atoms in each molecule are coplanar, the stacking mode of the three crystals is a planar layer style involving π···π stacking interactions and the three structures all show good safety performance. In addition, a large amount of inter- and intramolecular interaction exists in the three crystals. All principal types of intermolecular contacts in three crystals are N···H interactions, indicating that N···H interactions are the main driving force for the three crystals and they account for 40.9%, 38.9% and 32.9% in the three crystals, respectively. Apart from these weak interactions, there are hydrogen bonds, vdW interactions and steric effects in the three crystals, and these interactions in Crystal **c** are stronger than in Crystals **a** and **b**. In the ESP analysis, the distribution area of positive ESPs is significantly different for the three crystals, and the negative areas all concentrate on the nitrogen atoms of the molecules. Electrostatic potential also shows that the impact sensitivity and detonation velocity of the three crystals decreases in the sequence Crystal **c** > Crystal **a** > Crystal **b**. These results will prove meaningful in building heterocyclic ring energetic molecules in order to design and synthesize new EMs.

## Data Availability

Not applicable.

## Supplementary Materials

The following supporting information can be downloaded at: https://www.mdpi.com/article/10.3390/molecules27154969/s1, Table S1: The crystallographic parameters of three energetic crystals of the Existing CCDC.

## Sample Availability

Samples of the compounds are not available from the authors.
