# Supplementary material for: Crystal Structure and Noncovalent Interactions of Heterocyclic Energetic Molecules
Source: Molecules. 2022 Aug 4;27(15):4969. doi: 10.3390/molecules27154969 (PMC9370629; doi:10.3390/molecules27154969)
Supplement: Supplementary file 1 [file molecules-27-04969-s001.zip › molecules-1786162-supplementary.pdf]

Supplementary Materials

Crystal Structure and Noncovalent Interactions of Heterocyclic Energetic Molecules

Yan Liu [1,*], Jiake Fan [2], Zhongqing Xue [1], Yajing Lu [1], Jinan Zhao [1] and Wenyan Hui [1]

[1] Department of Environmental and Safety Engineering, Taiyuan Institute of Technology, Taiyuan 030008

[2] School of Environment and Safety Engineering, North University of China,
Taiyuan 030051, China

* Correspondence: liuyan@tit.edu.cn

Table S1. The crystallographic parameters of three energetic crystals of the Existing CCDC.

**Compound**	**a**[1]	**b**[1]	**c**·H_2O[1]
Chemical formula	$C_8H_7N_5$	$C_6H_3N_7O$	$C_5H_6N_8O_3$
Formula weight	173.19	189.15	226.18
Temperature (K)	173.0	170.0	170.0
Crystal system	Triclinic	Monoclinic	Monoclinic
Space group	$P\bar{1}$	$P2_1/c$	$P2_1/c$
a (Å)	6.8022(12)	9.8196(7)	6.7334(2)
b (Å)	7.0362(15)	11.6192(7)	15.5167(6)
c (Å)	8.8798(16)	6.8836(4)	8.6401(3)
α (°)	74.652(7)	90	90
β (°)	77.599(6)	103.218	110.321(1)
γ (°)	71.071(7)	90	90
Unit-cell volume (Å^3)	383.79(13)	764.58(8)	846.54(5)
Z	2	4	4
Density (g·cm^{-3})	1.499	1.643	1.77

References

1. Liu, Y.-J.; Qi, X.-J.; Zhang, W.-Q.; Yin, P.; Cai, Z.-W.; Zhang, Q.-H. Construction of bicyclic 1,2,3-triazine *N*-oxides from aminocyanides. *Org. Lett.* **2021**, *23*, 734–738.
